# Supplementary material for: Carbapenem-Resistant Gram-Negative Bacteria-Related Healthcare-Associated Ventriculitis and Meningitis: Antimicrobial Resistance of the Pathogens, Treatment, and Outcome
Source: Microbiol Spectr. 2022 Apr 25;10(3):e00253-22. doi: 10.1128/spectrum.00253-22 (PMC9241620; doi:10.1128/spectrum.00253-22)
Supplement: SUPPLEMENTAL FILE 1 — Supplemental material. Download spectrum.00253-22-s001.pdf, PDF file, 0.1 MB [file spectrum.00253-22-s001.pdf]

Appendix 1. Antimicrobial agents used in antimicrobial susceptibility tests and n (%) of resistant isolators in the nine years.

	2012	2013	2014	2015	2016	2017	2018	2019	2020
Total	3	12	6	4	8	13	16	17	13
Meropenem	3	12	6	3	8	13	15	13	10
Resistant, n (%)	1 (33.3)	10 (83.3)	4 (66.7)	2 (66.7)	8 (100.0)	12 (92.3)	10 (66.7)	12 (92.3)	9 (90.0)
Imipenem	3	12	6	4	8	13	16	17	13
Resistant, n (%)	3 (100.0)	11 (91.7)	6 (100.0)	4 (100.0)	7 (87.5)	13 (100.0)	16 (100.0)	17 (100.0)	13 (100.0)
Amoxicillin-clavulanate	2	3	3	2	2	3	1	4	6
Resistant, n (%)	2 (100.0)	2 (66.7)	3 (100.0)	2 (100.0)	2 (100.0)	3 (100.0)	1 (100.0)	4 (100.0)	6 (100.0)
Ampicillin-sulbactam	3	8	4	3	7	11	14	15	4
Resistant, n (%)	3 (100.0)	6 (75.0)	4 (100.0)	3 (100.0)	5 (71.4)	9 (81.8)	9 (64.3)	12 (80.0)	4 (100.0)
Ciprofloxacin	3	12	6	4	8	13	16	16	8
Resistant, n (%)	1 (33.3)	10 (83.3)	4 (66.7)	3 (75.0)	8 (100.0)	11 (84.6)	11 (68.8)	14 (87.5)	7 (87.5)
Amikacin	3	12	6	4	8	13	15	9	9
Resistant, n (%)	1 (33.3)	8 (66.7)	4 (66.7)	0 (0.0)	6 (75.0)	8 (61.5)	7 (46.7)	2 (22.2)	6 (66.7)
Aztreonam	2	7	5	4	3	7	11	7	5
Resistant, n (%)	1 (50.0)	5 (71.4)	4 (80.0)	3 (75.0)	3 (100.0)	6 (85.7)	6 (54.5)	6 (85.7)	5 (100.0)
Piperacillin	3	12	6	3	8	13	15	7	
Resistant, n (%)	1 (33.3)	9 (75.0)	5 (83.3)	2 (66.7)	7 (87.5)	12 (92.3)	10 (66.7)	5 (71.4)	
Piperacillin-tazobactam	3	12	6	4	7	13	15	14	13
Resistant, n (%)	1 (33.3)	9 (75.0)	4 (66.7)	3 (75.0)	7 (100.0)	12 (92.3)	8 (53.3)	11 (78.6)	12 (92.3)
Gentamicin	3	12	6	4	8	13	16	16	
Resistant, n (%)	1 (33.3)	9 (75.0)	5 (83.3)	0 (0.0)	6 (75.0)	10 (76.9)	11 (68.8)	14 (87.5)	
Cefepime	3	12	6	4	8	13	16	17	13
Resistant, n (%)	1 (33.3)	10 (83.3)	4 (66.7)	3 (75.0)	8 (100.0)	12 (92.3)	11 (68.8)	16 (94.1)	11 (84.6)
Cefotaxime	3	9	5	2	8	9	1		
Resistant, n (%)	2 (66.7)	8 (88.9)	4 (80.0)	2 (100.0)	8 (100.0)	9 (100.0)	1 (100.0)		
Ceftazidime	3	12	6	4	8	13	16	17	13
Resistant, n (%)	2 (66.7)	10 (83.3)	5 (83.3)	3 (75.0)	8 (100.0)	12 (92.3)	11 (68.8)	16 (94.1)	12 (92.3)
Levofloxacin	3	12	6	4	8	13	16	17	13
Resistant, n (%)	1 (33.3)	9 (75.0)	4 (66.7)	3 (75.0)	7 (87.5)	10 (76.9)	6 (37.5)	9 (52.9)	10 (76.9)
Polymyxin	3	12	6	3	8	10	1		
Resistant, n (%)	1 (33.3)	2 (16.7)	1 (16.7)	0 (0.0)	1 (12.5)	0 (0.0)	0 (0.0)		
Ampicillin	2	3	3	3	2	5			
Resistant, n (%)	2 (100.0)	2 (66.7)	3 (100.0)	3 (100.0)	2 (100.0)	5 (100.0)			
Trimethoprim-sulfamethoxazole	3	9	5	3	8	12	14	17	12
Resistant, n (%)	1 (33.3)	2 (22.2)	1 (20.0)	0 (0.0)	6 (75.0)	7 (58.3)	7 (50.0)	5 (29.4)	2 (16.7)
Tetracycline	3	9	4	2	7	9	1		

Resistant, n (%)	1 (33.3)	6 (66.7)	2 (50.0)	0 (0.0)	6 (85.7)	6 (66.7)	1 (100.0)		
Chloramphenicol	2	4	4	2	3	3	1		
Resistant, n (%)	0 (0.0)	2 (50.0)	1 (25.0)	0 (0.0)	0 (0.0)	0 (0.0)	1 (100.0)		
Ceftizoxime	2	4	4	3	3	6	9	7	
Resistant, n (%)	2 (100.0)	3 (75.0)	3 (75.0)	3 (100.0)	2 (66.7)	6 (100.0)	9 (100.0)	7 (100.0)	
Cefoxitin								4	6
Resistant, n (%)								4 (100.0)	6 (100.0)
Tobramycin			2	1		4	16	16	10
Resistant, n (%)			2 (100.0)	0 (0.0)		2 (50.0)	11 (68.8	13 (81.3)	8 (80.0)
Nitrofurantoin			2	1		4	16	16	4
Resistant, n (%)			2 (100.0)	1 (100.0)		3 (75.0)	15 (93.8)	14 (87.5)	4 (100.0)
Ceftriaxone							8	17	8
Resistant, n (%)							7 (87.5)	16 (94.1)	8 (100.0)
Cefuroxime							8	17	8
Resistant, n (%)							8 (100.0)	16 (94.1)	8 (100.0)
Cefotetan							8	16	
Resistant, n (%)							7 (87.5)	15 (93.8)	

Appendix 2. The antimicrobial resistance and treatment of different groups

	Group A<br>(n=41)	Group B<br>(n=12)	Group C<br>(n=39)
Initial empirical treatment	Meropenem<br>(n=30)<br>Trimethoprim-sulfamethoxazole<br>(n=2)<br>Ceftazidime<br>(n=2)<br>Piperacillin sulbactam<br>(n=2)<br>Cefoperazone Sulbactam<br>(n=1)<br>Multiple<br>(n=1)	Meropenem<br>(n=9)<br>Levofloxacin<br>(n=1)<br>Ceftazidime<br>(n=1)<br>Multiple<br>(n=1)	Meropenem<br>(n=35)<br>Meropenem/sulbactam<br>(n=3)<br>Etimicin<br>(n=1)
Judgment	Active<br>(n=16)<br>Untested<br>(n=1)<br>Inactive<br>(n=19)	Inactive<br>(n=10)	Same-type active<br>(n=1)<br>Other untested<br>(n=3)<br>Same-type inactive<br>(n=9)<br>Inactive<br>(n=26)
Adjusted empirical treatment	Piperacillin sulbactam to meropenem<br>(n=2)<br>Meropenem to etimicin<br>(n=1)<br>Meropenem to cefoperazone sulbactam<br>(n=1)<br>Meropenem to trimethoprim-sulfamethoxazole<br>(n=1)	Levofloxacin+ceftazidime to meropenem<br>(n=1)	Meropenem to tigecycline<br>(n=3)<br>Meropenem to Meropenem/sulbactam<br>(n=2)<br>Meropenem to polymyxin<br>(n=2)<br>Meropenem to cefoperazone sulbactam<br>(n=1)
Judgment	Active<br>(n=17)<br>Other untested<br>(n=1)<br>Same-type inactive<br>(n=1)<br>Inactive	Inactive<br>(n=10)	Same-type active<br>(n=2)<br>Other untested<br>(n=10)<br>Same-type inactive<br>(n=7)<br>Inactive

	(n=17)		(n=20)
Initial targeted treatment	Meropenem<br>(n=20)<br>Trimethoprim-sulfamethoxazole<br>(n=10)<br>Levofloxacin<br>(n=2)<br>Cefoperazone sulbactam<br>(n=2)<br>Piperacillin sulbactam<br>(n=1)<br>Piperacillin tazobactam<br>(n=1)<br>Ceftazidime<br>(n=1)<br>Etimicin<br>(n=1)<br>Aztreonam<br>(n=1)<br>Meropenem/sulbactam<br>(n=1)<br>Multiple<br>(n=1)	Meropenem<br>(n=6)<br>Piperacillin sulbactam<br>(n=1)<br>Levofloxacin<br>(n=1)<br>Ceftazidime<br>(n=2)<br>Multiple<br>(n=3)	Meropenem<br>(n=12)<br>Meropenem/sulbactam<br>(n=11)<br>Tigecycline<br>(n=6)<br>Cefoperazone sulbactam<br>(n=3)<br>Etimicin<br>(n=3)<br>Polymyxin<br>(n=2)<br>Levofloxacin<br>(n=1)<br>Multiple<br>(n=2)
Judgement	Active<br>(n=29)<br>Same-type active<br>(n=1)<br>Other untested<br>(n=3)<br>Inactive<br>(n=5)	Inactive<br>(n=7)	Same-type active<br>(n=3)<br>Other untested<br>(n=21)<br>Same-type inactive<br>(n=4)<br>Inactive<br>(n=8)
Adjusted targeted treatment	Meropenem to polymyxin<br>(n=3)<br>Meropenem to Trimethoprim-sulfamethoxazole<br>(n=2)<br>Meropenem to Trimethoprim-sulfamethoxazole+polymyxin<br>(n=1)	-	Meropenem to polymyxin<br>(n=4)<br>Meropenem to tigecycline<br>(n=2)<br>Meropenem to tigecycline+ etimicin<br>(n=1)<br>Meropenem to etimicin<br>(n=1)<br>Meropenem to cefoperazone sulbactam+meropenem/

	Trimethoprim-sulfamethoxazole to tigecycline<br>(n=1)<br>Meropenem to levofloxacin<br>(n=1)<br>Meropenem to ceftazidime<br>(n=1)<br>Etimicin to polymyxin+tigecycline<br>(n=1)<br>Cefoperazone sulbactam to polymyxin<br>(n=2)<br>Meropenem/sulbactam to polymyxin<br>(n=1)		sulbactam+levofloxacin<br>(n=1)<br>Meropenem/sulbactam to polymyxin<br>(n=2)<br>Meropenem/sulbactam to cefoperazone sulbactam<br>(n=1)<br>Meropenem/sulbactam to etimicin<br>(n=1)<br>Tigecycline to polymyxin<br>(n=2)<br>Tigecycline to cefoperazone Sulbactam<br>(n=1)<br>Add etimicin<br>(n=1)
Judgment	Active<br>(n=39)	Inactive<br>(n=7)	Same-type active<br>(n=6)<br>Other untested<br>(n=28)<br>Same-type inactive<br>(n=3)

Group A: Cases received active antimicrobial agents as the antimicrobial agents with the highest priority;

Group B: Cases received inactive antimicrobial agents as the antimicrobial agents with the highest priority;

Group C: Cases received untested antimicrobial agents as the antimicrobial agents with the highest priority.

Same-type active or inactive: meropenem and imipenem, amikacin and etimicin, and tetracycline and tigecycline were seen as same-type antimicrobial agents in this study, respectively. If an antimicrobial agent was active or inactive, and another antimicrobial agent was untested, the another antimicrobial agent was seen as a same-type active or

inactive antimicrobial agent.
